# Protective Effect of Sodium Nitroprusside on the Rat Small Intestine Transplanted Mucosa

**DOI:** 10.1155/2015/786010

**Published:** 2015-01-11

**Authors:** Feng-Hua Chen, Ke Li, Lu Yin, Chun-Qiu Chen, Zhao-Wen Yan, Gui-Ming Chen

**Affiliations:** ^1^Ultrasound Department, Obstetrics and Gynecology Hospital of Fudan University, Shanghai 200090, China; ^2^Department of General Surgery, Shanghai Jiaotong University Affiliated First People's Hospital, Shanghai 200080, China; ^3^Shanghai Institute of Digestive Surgery, Shanghai Jiaotong University Medical College, Shanghai 200025, China; ^4^Department of Pathology, Shanghai Jiaotong University Medical College, Shanghai 200025, China

## Abstract

The intestinal mucosal epithelium is extremely susceptible to even brief periods of ischemia. Mucosal barrier damage, which is associated with ischemia/reperfusion (I/R) injury and consequently bacterial translocation, remains a major obstacle for clinically successful small bowel transplantation (SBT). Previous studies have demonstrated a protective effect of nitric oxide (NO) on other transplanted organs and NO mediated intestinal protection has also been reported* in vitro*. The aim of this study was to evaluate the effect of sodium nitroprusside (SNP), NO donor, on graft mucosal histology and molecular markers of function after SBT in rats. We used SNP in different period of heterotopic SBT rats. The groups consisted of SBT, pre-SNP group, and post-SNP group. Interestingly, the pre-SNP graft samples exhibited less damage compared to the SBT and post-SNP samples. In addition, mucosal samples from the pre-SNP group showed higher Na^+^-K^+^-ATPase activity and higher levels of laminin expression compared to the SBT and post-SNP samples. The findings of the present study reveal that SNP given before graft ischemia/reperfusion injury has a protective effect on mucosal histology and molecular markers of function in the transplanted small intestine.

## 1. Introduction

Small bowel transplantation (SBT) is the only definitive treatment for patients with end-stage intestinal failure. While new immunosuppressive drugs and advanced surgical procedures have greatly improved the clinical outcome, major obstacles continue to exist for the success of SBT. Nevertheless, SBT still remains the intervention of second choice after total parenteral nutrition (TPN) under most conditions. The two most significant obstacles are bacterial infection and allograft rejection with 67% of patient mortality attributable to sepsis (55%) or rejection (12%) [1]. After transplantation, sepsis is the direct consequence of bacterial translocation across the injured mucosal barrier of the small bowel grafts. In addition, the injured graft aggravates allograft rejection because of submucous tissue antigen exposure [2]. Therefore, treatment strategies to protect the graft's mucosal barrier during transplantation are a key factor in the ongoing efforts towards clinically successful transplantation of the small bowel.

Current literature indicates that intracoronary administration of the NO donor, S-nitroso-N-acetyl-D,L-penicillamine (SNP), for a brief period before ischemia can reduce infarct size, attenuate neutrophil accumulation, and improve endothelial function [3]. A number of studies have also demonstrated that exogenously administered nitric oxide (NO) can alleviate experimental liver, lung, intestine, and renal I/R injury, suggesting that NO can play an important role in attenuating tissue injury during the reperfusion phase [4–7]. Based on these previous studies, we have designed experiments to explore the therapeutic potential of exogenous NO in the context of SBT in a rat model.

## 2. Materials and Methods

### 2.1. Animals and Reagents

Male Sprague-Dawley (SD) rats (220~300 g) were obtained from Shanghai Songlian Laboratory Animal Center (Shanghai, China). The study was approved by the regional ethical committee for laboratory animal experiments and all experiments were conducted in accordance with the regulations and policies of the Chinese Council on Animal Care. Animals were fasted overnight and housed in wire-bottomed cages to prevent coprophagy. Water was freely available. Surgical procedures were performed on rats anesthetized by diethyl ether and maintained by intermittent ether supplement.

All chemicals were AR-grade. Coomassie Blue staining kit was obtained from Nanjing Jiancheng Bioengineering Research Institute (Nanjing, China). PCR primers were designed and synthesized according to the* Rattus* laminin cDNA sequence by Shanghai Sagon (Shanghai, China). RT kit and HotStarTaq Master Mix were purchased from QIAGEN (Düsseldorf, Germany); Trizol Reagent was obtained from Invitrogen (California, USA). Immunohistochemical and* in situ* hybridization kits were from BOSTER (Nanjing, China), and SNP was purchased from Sigma (Shanghai, China).

### 2.2. Experimental Groups

Eighty-four male Sprague-Dawley (SD) rats weighing 220~300 g were randomly assigned as donor or recipient to construct an established heterotopic SBT (H-SBT) model. Animals were allocated to three groups. The heterotopic transplanted groups consisted of (1) SBT with no SNP treatment (*N* = 12) and (2) pre-SNP group (*N* = 14), in which treatment consisted of SNP injection into the penile vein at three time points (6, 3, and 0 hours) before donor organ harvest and heterotopic engraftment. Briefly, SNP was dissolved in lactated-ringers (LR) solution and given as a bolus dose of 5 *μ*g (5 *μ*g/0.5 mL). (3) Post-SNP group (*N* = 16), in which treatment consisted of penile vein injection of SNP (5 *μ*g/0.5 mL) after heterotopic SBT at 0, 3, and 6 hours after engraftment. Recipients surviving 72 hours after operation with a successful stoma were used for experimental analysis.

### 2.3. Surgical Procedure for Heterotopic Small Bowel Transplantation

**Figure 1:**
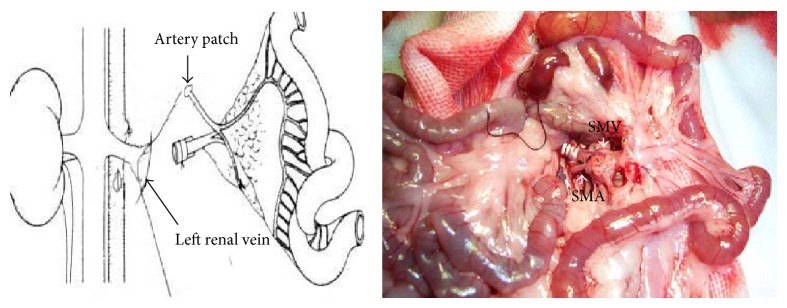
*Vessel reconstruction*: donor graft's cuffed portal vein is inserted into left renal vein of the recipient and an end to side anastomosis is performed between SMA and the abdominal aorta. The right picture shows a typical graft 20 seconds after artery anastomosis and vein insertion and demonstrates good blood circulation in the graft.

The H-SBT model was based on the modified classical method [8] and performed consistently by the same surgical team. The cuff technique was used for venous reconstruction. Briefly, the donor small bowel vasculature was perfused with LR solution through the superior mesenteric aorta* in vivo* after branch ligation and clamping of the superior and inferior aorta leaving the portal vein patent. Twenty centimeters of small bowel 5 cm distal to the ligament of Treitz isolated on the superior mesenteric vascular pedicle attached to stumps of the aorta and portal vein was used for the graft. After harvest, the intestinal lumen was gently cleared with LR solution and the graft was stored on ice at 4°C until transplantation. The donor portal vein was cuffed (a manual cuff was made using a 2 mm DSA tube) and ligated to the recipient left kidney vein (left kidney was resected) to reconstruct venous outflow, and an aortic patch was anastomosed end to side to the recipient infrarenal aorta by running suture to reconstruct arterial inflow. The oral end of the transplanted intestine was closed and the distal end exteriorized through the left lower abdominal wall creating a cutaneous stoma (Figure 1). Intestinal samples for molecular analysis were harvested 72 hours after transplantation through the graft stoma, frozen in liquid nitrogen, and stored at −80°C until further use.

### 2.4. Histology

Full thickness graft samples harvested 72 hours after transplantation were fixed in 10% buffered formalin, embedded in paraffin, cut to 4-5 *μ*m thick sections, and stained with hematoxylin-eosin. Histological damage was graded using Park's histologic classification of intestinal tissue injury by a blinded pathologist [8]. The samples were scored as follows: 0: normal mucosa; 1: subepithelial space; 2: extended subepithelial space; 3: epithelial lifting along villus side; 4: denuded villi; 5: loss of villus tissue; 6: crypt layer infarction; 7: transmucosal infarction; and 8: transmural infarction.

### 2.5. Graft Mucosal Na^+^-K^+^-ATPase Activity

Na^+^-K^+^-ATPase activity was analyzed using a Na^+^-K^+^-ATPase detection kit according to the instructions of the manufacturer (Nanjing Jiancheng Bioengineering Research Institute, China). Briefly, frozen intestinal samples were thawed and diluted with 10 volumes of physiological saline to make a 10% homogenate. Then, 9 volumes of physiological saline were added to the homogenate to make 1% homogenate just before detection. The samples were homogenized with an Ultra-Turrax homogenizer (Labassco, Sweden). The homogenates were centrifuged at 5000 g for 30 min at 2°C. The absorbance was read on a spectrophotometer (Victor 2, Wallac, Sweden) at 636 nm. Spectrophotometric measurements were normalized with sample tissue protein levels detected by Coomassie Blue staining. Graft Na^+^-K^+^-ATPase activity was expressed in units/mg (protein)/h. One unit of Na^+^-K^+^-ATPase is defined as that quantity of enzyme which hydrolyzes 1 *μ*mol ATP in one hour at 25°C.

### 2.6. Laminin Expression

Graft laminin expression was quantified in each sample by semiquantitative RT-PCR* in situ* hybridization and protein immunohistochemistry according to the manufacturer's protocol. The following primers were used.

Laminin primer sequences:

 5′-GTGTCTTCAGAGGTGACTGTATTCG-3′,
 5′-TTCTCCCGGTTCTTGATGCT-3′,

*β*-actin primer sequences:

 5′-ACATCTGCTGGAAGGTCCAC-3′,
 5′-GTACCACCATGTACCCAGG-3′.

### 2.7. Statistical Analysis

Na^+^-K^+^-ATPase activities are presented as mean ± SE. Differences were tested for statistical significance by Newman-Keuls ANOVA. *P* < 0.05 was considered statistically significant. Recipient and stoma mortality were analyzed by exact probability method. All statistical analyses were performed using SAS9.3 software.

## 3. Results

### 3.1. Recipient Mortality Rate

**Figure 2:**
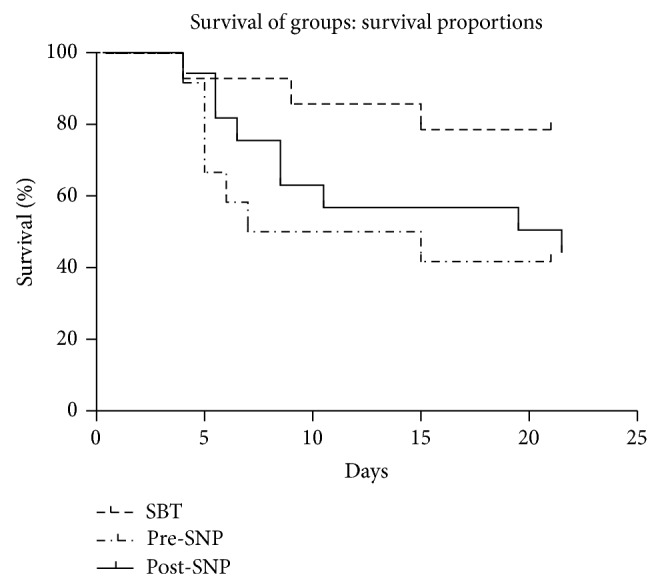
*Survival Curve of three groups*: a significantly lower mortality rate in the pre-SNP group (*n* = 14) compared to that in SBT group (*n* = 12) and post-SNP group (*n* = 16) during 3 weeks of observation (*P* < 0.05).

We observed a significantly lower mortality rate in the pre-SNP group (21.4%) compared to the SBT group (58.3%) and post-SNP group (56.3%) during the 3 weeks of observation (*P* < 0.05). There was no difference in survival between the SBT group and post-SNP group (*P* > 0.05). Interestingly, most animals died within 10 days after transplantation in the SBT and post-SNP groups. Survival rate was relatively stable in all groups after 2 weeks (Figure 2).

### 3.2. Histopathology

Tissue damage was evaluated 72 hours after intestinal transplantation in accordance with Park's classification system. Specimens that were scored 3 to 6 were classified as stoma morbidity and those that scored 7 or 8 were classified as stoma mortality.

Similar to morbidity of recipients, stoma morbidity was significantly lower in the pre-SNP group compared to the SBT group and post-SNP group (*P* < 0.05). There was no difference in survival between the SBT and post-SNP groups within 10 days after transplantation (*P* > 0.05) (Table 1).

**Figure 3:**
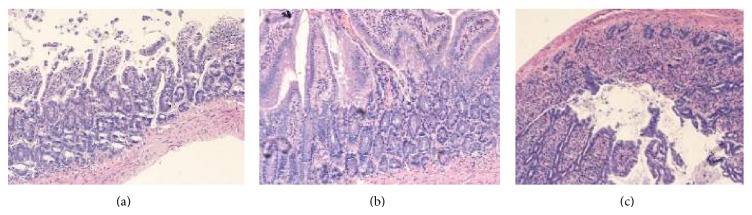
Histological examination of the graft 72 hours after SBT. (a) A representative sample from the SBT group (*n* = 12): obvious intestinal villi atrophy, destroyed villi, denuded villi, dilated capillaries, and some crypt layer injury. (b) A representative sample from the pre-SNP group (*n* = 14): some mild epithelial injuries are present. (c) Post-SNP group (*n* = 16): obvious destroyed, atrophic, and denuded villi and dilated capillaries are visible and some crypt layer injuries are present (HE ×200).

As expected, graft implantation and reperfusion resulted in significant injury to the mucosal architecture in the SBT group. However, near normal mucosal architecture with only minimal injury and decreased villus length was observed in the pre-SNP group (Figure 3). In contrast, a higher degree of histological injury was found in the post-SNP group, in which samples exhibited massive villus lifting and exposure of the subepithelial space.

### 3.3. Graft Mucosal Na^+^-K^+^-ATPase Activity

**Figure 4:**
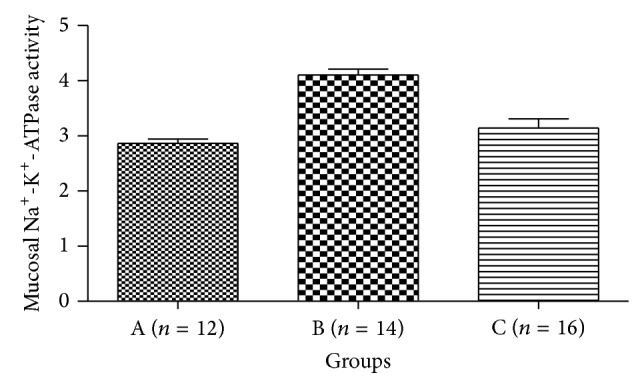
Graft mucosal Na^+^-K^+^-ATPase activity. In the pre-SNP group (B) Na^+^-K^+^-ATPase activity was significantly higher than that in the SBT group (A) (*P* < 0.05). There is no difference between post-SNP group (C) and the SBT group (A) (*P* > 0.05).

Average Na^+^-K^+^-ATPase activity of graft mucous membrane in grafts pre-SNP group (4.106 ± 0.3957 U/mg) harvested 72 hours after transplantation was significantly higher than that in the SBT group (2.867 ± 0.2741 U/mg) (*P* < 0.05). There was no difference between the post-SNP (3.1425 ± 0.6664 U/mg) and the SBT groups (Figure 4).

### 3.4. Laminin Expression

**Figure 5:**
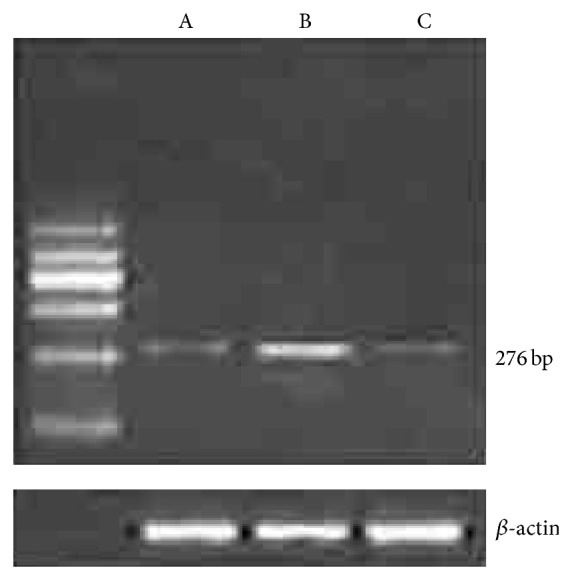
Semiquantitative RT-PCR analysis of laminin mRNA expression. Significantly higher laminin mRNA expression was observed in the pre-SNP group (B) compared to SBT (A) and post-SNP group (C).

**Figure 6:**
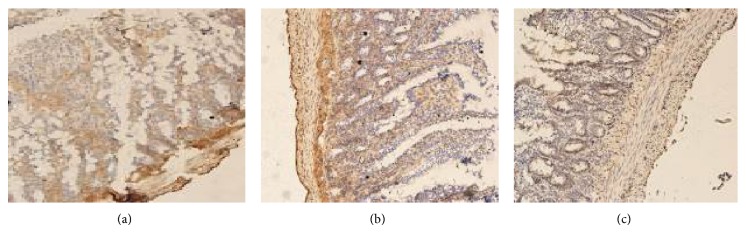
Laminin protein expression. A representative sample from the SBT group (*n* = 12): an absence of mucosal laminin protein expression and severe injury of intestinal villi are present. (b) A representative sample from the pre-SNP group (*n* = 14): lower mucosal laminin protein expression and mild injury of intestinal villi are observed. (c) Post-SNP group (*n* = 16): absence of mucosal laminin protein expression and severe injury of intestinal villi are observed (×200).

**Figure 7:**
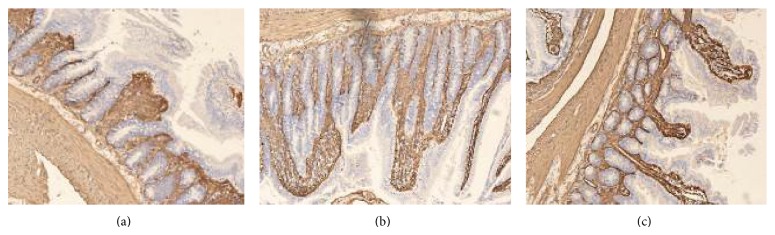
Lamininhybridization* in situ*. Mucosal laminin mRNA is expressed in the basal part of the intestinal mucosa. A representative sample from the SBT group (*n* = 12): mucosal laminin mRNA expression is low with sections of an interrupted expression. (b) A representative sample from the pre-SNP group (*n* = 14): the expression is continuous. (c) Post-SNP group (*n* = 16): the expression is low and there are interruptions present (×200).

As shown in Figures 5, 6, and 7, the pre-SNP group exhibited a higher expression level of laminin as quantified by semiquantitative RT-PCR* in situ* hybridization and protein immunohistochemistry compared to the SBT and post-SNP groups. Laminin protein expression examined by immunohistochemistry was stronger in the pre-SNP group compared to the SBT and post-SNP groups. Experiments with hybridization and immunohistochemistry revealed that the laminin expression was localized to the mucosal epithelial basement membrane. There was no difference in laminin expression between the SBT and post-SNP groups.

## 4. Discussion

Small bowel transplantation could be the definitive procedure for the treatment of intestinal failure disease especially in settings where TPN is not tolerated. However, graft intestinal mucosa damage-related sepsis induced by bacterial translocation remains a major problem in the postoperative management of these patients and remains a crucial cause of death and graft loss [9]. A better understanding of the interactions between the inherent intestinal immune system and mucosa injury during I/R would be of benefit in developing novel treatment strategies. In addition, this knowledge would significantly lower the immunogenicity and hence improve the survival of the transplanted intestinal graft.

In the present study, we have utilized a heterotopic SBT rat model to investigate the therapeutic potential of NO in SBT. In this heterotopic model, the graft condition is easily observed and samples can be harvested from the stoma allowing longitudinal studies. Furthermore, the recipients eat freely soon after transplantation and have faster recovery due to reduced disturbance of the host's bowel.

In normal conditions, the mucosa of the adult bowel presents a balance between cells that proliferate and undergo apoptosis or necrosis. The mechanisms and mediators regulating these processes are currently under study. The intestinal mucosa is very sensitive to oxidative stress. SBT is one of the major clinical circumstances in which I/R induced oxidative stress plays an important role in inducing mucosal damage which can then lead to bacterial translocation [10]. Ischemia/reperfusion would be expected to cause an increase in mucosal permeability, resulting in a “bare area” of compromised barrier function where bacterial toxins or bacteria could attach, translocate, and provoke endotoxemia with the subsequent development of systemic inflammatory response syndrome [11]. It is known that structural mucosal barrier loss and intestine immune activity are responsible for this increase in functional permeability and bacterial translocation [12].

Ischemia/reperfusion injury to the intestinal mucosal barrier of the graft is nonspecific. The crypt cell epimatrix is the microstructural base station of the mucosal barrier, which is required for mucosa cell regeneration and cell differentiation [13]. Laminin is one of the most important elements in the cell epimatrix. It has multiple biological functions at the intestinal basal membrane and is associated with collagen networks. Synthesized by the basal epithelium, it modulates cell adhesion, migration, and differentiation, while secreted collagenases result in a dynamic balance of structural plasticity. The cellular epimatrix of the graft is an early focal target of I/R. Reduced synthesis and high levels of laminin degradation during I/R result in the attenuation and even breakdown of the basal membrane [14–16]. In a sense, laminin is essential to the integrity of the intestinal mucosal barrier, and, as it was demonstrated in our rodent model, an important indicator of graft damage after transplantation.

The involvement of NO in I/R is still controversial. Some authors have shown that the exogenous administration of NO attenuates postischemic lesions in some organs and tissues [3–5]. This reduction may be caused by NO's protective role against microvascular dysfunction and against leukocyte adhesion, key factors associated with postischemic reperfusion [4–7, 17]. However, other studies have indicated that excessive production of NO or pharmacologic administration of NO induces a reaction with superoxide anion, generating peroxynitrite, a highly reactive molecule involved in a wide variety of pathological reactions [18, 19]. Hence, the NO dose and species differences may contribute to the varied effects of exogenous NO.

Based on the data presented in the literature, the aim of this study was to explore the protective role of NO on the transplanted intestinal mucosa. Among the three different transplanted groups in the study, higher levels of laminin cDNA, mRNA, and protein expression were observed in the graft pre-SNP group compared to the SBT and post-SNP groups. Moreover, the results also showed that the levels of Na^+^-K^+^-ATPase activity were higher in the graft pre-SNP group compared to the SBT and post-SNP groups.

The fact that mucosal barrier integrity and good energy dynamic function were associated with successful SBT in the pre-SNP group indicates a potential therapeutic role for NO as a graft preconditioning reagent. Furthermore, our data showed that post-SNP treatment would not be beneficial.

In conclusion, the exogenous administration of NO via donor graft treatment improved the histology and energy reserve of the bowel graft as a preconditioning reagent but was not beneficial as a therapeutic reagent after transplantation. It is reasonable to assume that the protective effect of SNP was not present during the whole SBT procedure. These findings open up interesting possibilities for research into new feasible agents that may improve graft and recipient tissue viability in clinical SBT.

## Figures and Tables

**Table 1:** Stoma morbidity and mortality.

Group	*N*	Stoma		
		Morbidity (*n*)	Mortality (*n*)	Total (*n*, %)
SBT group	12	3	8	11 (91.7%)
Pre-SNP group	14	2	6	8 (57.1%)
Post-SNP group	16	5	9	14 (87.5%)
